# Supplementary material for: Development of a Nanoparticle-Based Approach for the Blood–Brain Barrier Passage in a Murine Model of Amyotrophic Lateral Sclerosis
Source: Cells. 2022 Dec 10;11(24):4003. doi: 10.3390/cells11244003 (PMC9776960; doi:10.3390/cells11244003)
Supplement: Supplementary file 1 [file cells-11-04003-s001.zip › cells-2050388-supplementary.pdf]

Development of a nanoparticle-based approach for the blood-brain barrier passage in a murine model of amyotrophic lateral sclerosis

Supplementary Material

1. Biotin-derivatives for ANANAS decoration

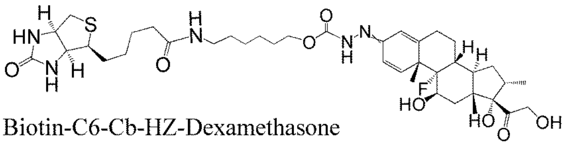

Biotin-C6-Cb-HZ-Dexamethasone

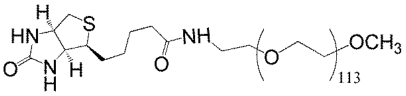

Biotin-methoxyPEG5kDa

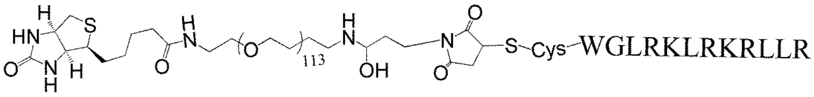

Biotin-PEG5kDa-Cys-ApoE

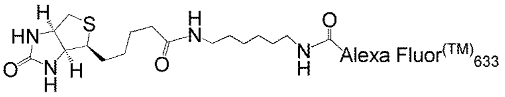

Biotin-C6-Alexa633

Figure S 1: *Chemical structures of the biotin derivatives used for ANANAS decoration*

2. ApoE peptide purification

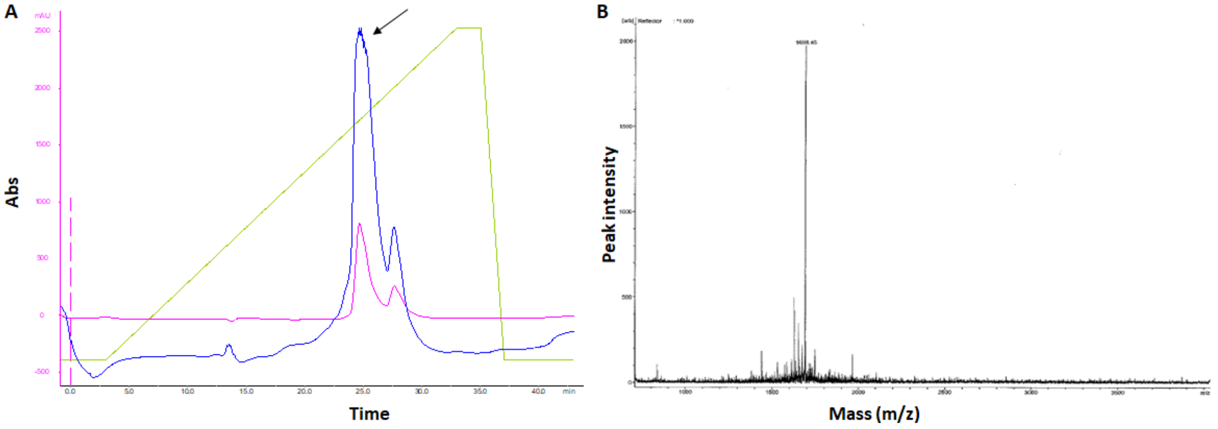

Figure S 2: *After synthesis, ApoE peptide was purified by reverse phase high performance liquid chromatography (HPLC) on a semi-preparative C4 column. Panel A shows the absorbance of the injected peptide (6 mg/ml) at 214 nm (blue, amide bound) and 280 nm (red, tryptophan absorption), the arrow indicates the peak corresponding to the peptide. The molecular weight of the sequence was verified using a MALDI-TOF mass spectrometer in reflector mode (Panel, B).*

3. ANANAS-ApoE pre-formulation studies

In the case of the ANANAS-ApoE assemblies, pre-formulation studies were carried out to assess the NP maximum loading capacity for biotin-Cys-ApoE and the colloidal stability of the formulations as a function of the ApoE load. To this end, core ANANAS were dissolved in 10 mM phosphate, 150 mM NaCl, pH 7.4 (PBS buffer) +0.1% BSA and mixed with biotin-Cys-ApoE at ApoE/NP molar ratios between 0 to 1340, corresponding to theoretical % biotin binding site (%BBS) coverages between 0-100.

The NP surface saturation limit for the biotin-Cys-ApoE was measured indirectly by assessing the ability of ANANAS-ApoE assemblies generated at BBS coverage between 0 to 100% to further tether biotin-Horseradishperoxidase (b-HRP; ANANAS nanotech, code# B-H0101). In brief, different ANANAS-ApoE assemblies (BBS% 0, 2.5; 5.0; 10; 20; 40; 60; 100; 4 µg/mL) were immobilized (1.5 µL) onto two nitrocellulose membrane. After blocking with a 3% BSA in PBS, the membranes were incubated with b-HRP (1µg/mL in PBS +0.05% tween20, RT, 1h) or non biotinylated HRP (control). After extensive washing, membranes were incubated with the 3, 3'-diaminobenzidine (DAB) in the presence of H_2O_2. No signal was detected at BBS coverage above 40%. This value was estimated as the NP maximum loading capability for biotin-Cys-ApoE.

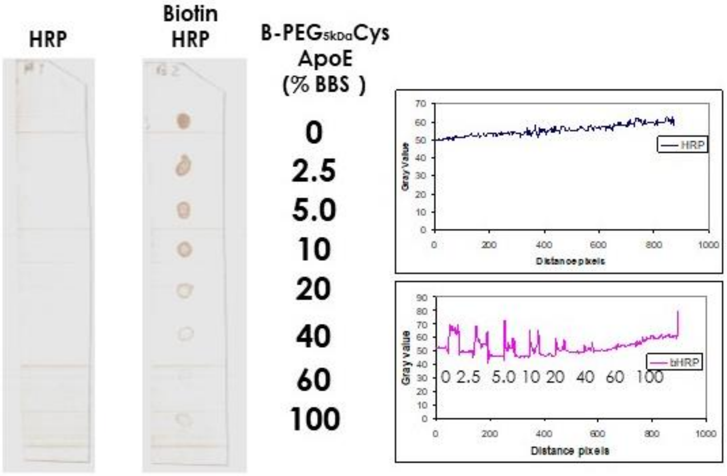

Figure S 3: *Dot blot assay on nitrocellulose membrane spotted with ANANAS/biotin-PEG-Cys-ApoE mixtures obtained at different ApoE:NP ratios*

The size of the ANANAS-ApoE assemblies generated at different ApoE:NP ratios was measured by dynamic light scattering one and 24h after assembly (to assess colloidal stability). Assemblies were colloidally stable up at a maximum 10% BBS coverage with the ApoE derivative.

Table S1. Pre-formulation data on ANANAS-ApoE assemblies. Core NPs were mixed in PBS+ 0,1% BSA with different molar ratios of biotin-PEG5kDa-Cys-ApoE. The size of the assemblies was measured 1 hour after preparation and after 24h storage at 4°C. The compositions highlighted in grey were judged unstable.

Formulation name	ApoE/NP (mol:mol)	Biotin-Cys-ApoE (% BBS)	Z-Average (nm) 1h	PDI (1h)	Z-Average (nm) 24h	PDI 24h
**ANANAS-ApoE0**	0.00	0.00	136.4±4.9	0.32	139.1±14.1	0.355
**ANANAS-ApoE2.5**	33.50	2.50	138.0±12.5	0.33	157.2±27.3	0.417
**ANANAS-ApoE5**	67.00	5.00	138.9±0.26	0.26	154.4±26.9	0.318
**ANANAS-ApoE10 (*)**	134.00	10.00	140.5±2.9	0.25	142.4±7.0	0.360
**ANANAS-ApoE20**	268.00	20.00	140.7±3.0	0.33	160.7±27.3	0.388
**ANANAS-ApoE40**	536.00	40.00	168.0±7.3	0.36	718.8±191.1	0.834
**ANANAS-ApoE60**	804.00	60.00	207.30±17.8	0.58	1,908.7±644.3	0.988
**ANANAS-ApoE100**	1,340.00	100.00	340.90±57.1	0.59	2,717.7±969.7	1.000

(*) selected for the in vivo studies

Since NP in vivo longitudinal tracking with the IVIS instrumentation necessitates the particles to be fluorescent, we performed additional pre-formulation studies to assess the impact of biotin-C6-alexa$_{633}$ on the colloidal stability of ApoE:ANANAS assemblies. Unfortunately, in the presence of the alexa$_{633}$ dye the nanoparticles loose solubility (not shown), likely because of non-specific interaction of the negatively charged dye with the highly positive charged ApoE moiety.

4. ANANAS-Dex pre-formulation studies

ANANAS-Dex was generated by mixing core NPs with sub-saturating (57.5% of BBS) amounts of B-C6-Cb-Hz-Dex. The lack of unbound conjugate in the assembly solution was verified by gel permeation chromatography by comparing the chromatogram of the low MW dexamethasone conjugate (two peaks - cis and trans Hz- with retention volume about 27 mL) before and after ANANAS (retention time about 8 mL) mixing (Figure S 4).

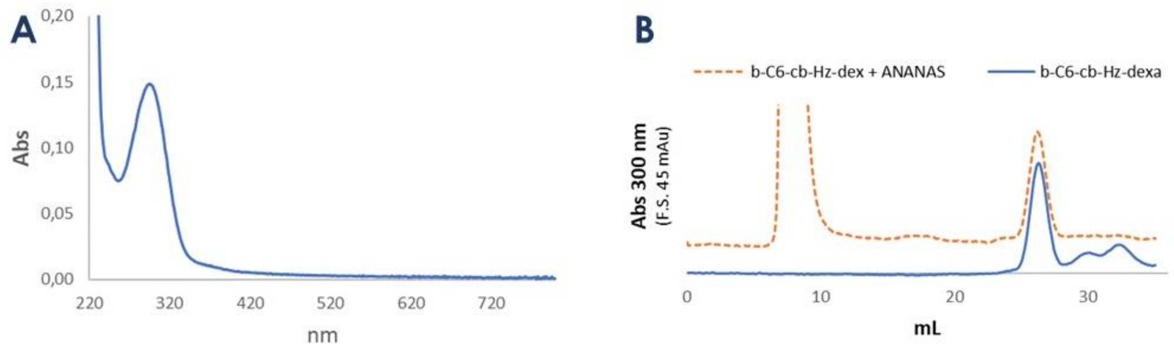

Figure S 4: *ANANAS-Dex preformulation study. A) UV-Vis spectrum of Biotin-C6-Cb-Hz-Dex (8x10^{-6}M in water) showing the characteristic dexamethasone-Hz conjugate absorbance maximum at 300 nm. B) Gel permeation chromatograms of biotin-C6-Cb-Hz-Dex as such and after addition of ANANAS core NPs (57.5% BBS coverage). Samples were eluted in a Superose prep™ medium, using the GE-Aktaflux- FPLC apparatus, PBS as eluant.*
